# Evidence for the Use of Isoflurane as a Replacement for Chloral Hydrate Anesthesia in Experimental Stroke: An Ethical Issue

**DOI:** 10.1155/2014/802539

**Published:** 2014-02-27

**Authors:** Pétrault Maud, Ouk Thavarak, Lachaud Cédrick, Bastide Michèle, Bérézowski Vincent, Pétrault Olivier, Bordet Régis

**Affiliations:** ^1^EA 1046-Département de Pharmacologie Médicale, Faculté de Médecine, CHU Lille, 1 Place de Verdun, 59045 Lille Cedex, France; ^2^UDSL, 59000 Lille, France; ^3^IUT A, Université de Lille 1, 59653 Villeneuve d'Ascq Cedex, France; ^4^Université d'Artois, 62307 Lens, France

## Abstract

Since an ethical issue has been raised regarding the use of the well-known anesthetic agent chloral hydrate, owing to its mutagenic and carcinogenic effects in animals, attention of neuroscientists has turned to finding out an alternative agent able to meet not only potency, safety, and analgesic efficacy, but also reduced neuroprotective effect for stroke research. The aim of this study was to compare the potential of chloral hydrate and isoflurane for both modulating the action of the experimental neuroprotectant MK801 and exerting analgesia. After middle cerebral artery occlusion in rats, no difference was observed in 24 h survival rate, success of ischemia, or infarct volume reduction between both anesthetics. However, isoflurane exerted a more pronounced analgesic effect than chloral hydrate as evidenced by formalin test 3 hours after anesthesia onset, thus encouraging the use of isoflurane in experimental stroke models.

## 1. Introduction

A cerebrovascular accident is defined by a sudden neurological deficit of vascular origin, as the damage of brain parenchyma arises from infarction (ischemic stroke) or bleeding (hemorrhagic stroke). Stroke is the third cause of death and the leading cause of acquired adult disability in the world, with still no neuroprotectant and few therapeutic options. This situation challenges the current knowledge of stroke pathophysiological events as well as the relevance of experimental models. This led researchers to develop several ischemic stroke models in a variety of species. To simulate human ischemic stroke, with 80% of which being thrombotic or embolic occurring in the territory of the middle cerebral artery (MCA), the intraluminal middle cerebral artery occlusion (MCAO) model has been set up and has been proved to be valuable in the field. This model requires a particularly efficient anesthesia of animals to induce stroke surgically. Indeed, it has both to last enough time for the microsurgery of the brain to be successful and to allow the awakening to be rapid. Moreover, in addition to safety, anesthesia of experimental stroke models has to be free of neuroprotective effect in order to avoid bias in the neuroprotection studies. Finally, for ethical reasons, the ideal anesthetic should provide postoperative analgesia since analgesics cannot be used in stroke model because of their potent neuroprotective action [1, 2].

To date, experimental anesthesia is based on an intraperitoneal injection of chloral hydrate since this method was confirmed as safe and realistic [3], with minimal effects on cardiovascular function or reflexes, as well as absence of synergistic neuroprotection in stroke studies [4]. However, when surgical anesthesia doses are administered, the safety margin is significantly reduced and recovery is prolonged [5]. Chloral hydrate metabolism has received increased attention in recent years as a metabolite of trichloroethylene, a metal-degreasing solvent found to be carcinogen in rodents [6, 7]. Moreover, hepatocarcinogenicity has been associated with the cardiopathic effect of high dose of chloral hydrate [8]. For these reasons, the French Agency for Safety of Health Products (AFSSAPS) has questioned the benefit/risk ratio of this compound in 2000, leading to possible withdraw from the market [9].

Isoflurane is a volatile halogenated gas that is currently used in stroke studies but also reported to exert neuroprotection [10, 11], the mechanisms of which are however poorly understood. Among numerous mechanisms, isoflurane may antagonize the NR1 subunit of the N-methyl-D-aspartate (NMDA) glutamate receptor, thus preventing its input on synaptic transmission [12, 13]. However, this anesthetic agent has very interesting advantages such as ease of use, titration, and rapid awakening which deserves interest in MCAO model.

To assess the ability of isoflurane and chloral hydrate to interfere with pharmacologically induced neuroprotection, we used MK801, a well-described NMDA receptor antagonist, used as a reference compound for experimental neuroprotection in rodents [14–16]. Since animal pain is centrally considered in the ethical issues of experimental studies, and therefore in the requirements for anesthetics, a pain assessment was included in the study in order to check for the postoperative analgesic potential of both compared anesthetics.

## 2. Experimental Procedure

### 2.1. Animals and Drugs Administration

All experiments were performed in accordance with the European Community Legislation (2010/63/UE). The Local Ethics Committee approved the experiments. All rats in this study were adult male Wistar (Janvier SAS, Le Genest-St-Isle, France) weighing 280–330 g according to MCAO model. Animals were housed under controlled laboratory conditions, with a 12-hour dark cycle, a temperature of 21 ± 2°C, and a humidity of 60 to 70%. The animals had *ad libitum* access to standard chow and tap water.

Two experimental protocols were conducted during this study. The first protocol aimed at studying the modulation of infarct volumes by both anesthetics (with or without MK801). The second protocol has examined the analgesic potential of these anesthetics through a pain test, the formalin test. This test was performed on either healthy animals or ischemic animals.

Two modes of anesthesia were tested (see Table 1). MK801 (SIGMA, M107; 0.5 mg/kg in saline) was injected intravenously 3 minutes before MCAO.

**Figure 1:**
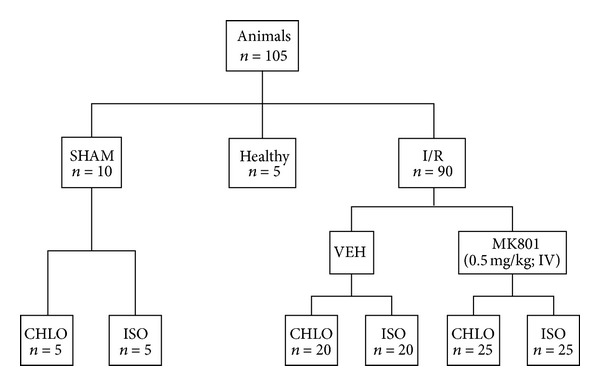
Experimental design and classification of animal groups. I/R: ischemia/reperfusion animals; VEH: vehicle group (saline); ISO: isoflurane anesthesia (4% induction, 2% maintenance); CHLO: chloral hydrate anesthesia (300 mg/kg ip). MK801 group corresponded to animals who received MK801 IV injection (0.5 mg/kg) 3 minutes before MCAO.

One hundred and five animals were included in the study and were randomly assigned to the different groups. Ninety animals have experienced cerebral ischemia (I/R groups): chloral (CHLO), *n* = 20; chloral with MK801 treatment (CHLO + MK), *n* = 25; isoflurane (ISO), *n* = 20; isoflurane with MK801 treatment (ISO + MK), *n* = 25. Ten animals were included in the SHAM group (operated animals without MCAO), *n* = 5 per group (CHLO and ISO). Five animals were included in the Healthy group for formalin test. Healthy animals underwent no anesthesia and no surgery and were considered as controls (Figure 1).

Animals that died at the end of the protocol or survived but without successful ischemia (i.e., without lesion or with only subcortical lesion) were excluded from the study.

### 2.2. The MCA Occlusion Model

After anesthesia, a rectal probe was inserted and body temperature was maintained at 37 ± 0.5°C with a heating lamp.

The ostium of the right MCA was occluded intraluminally, as previously described [17]. The right carotid arteries were exposed through a midline cervical incision and the common carotid and external carotid arteries were ligated with a silk suture. The pterygopalatine artery was exposed (by developing a plane alongside the internal carotid artery) and then ligated at its origin with a fine silk. An aneurysm clip was placed across internal carotid artery and an arteriotomy was made in the common carotid artery stump, allowing the introduction of a 4/0 monofilament nylon suture with its tip rounded by flame heating. Once the suture was in place, the aneurysm clip on the internal carotid artery was removed. The suture was gently advanced into the internal carotid artery and passed into the intracranial circulatory system to lodge in the narrower lumen of the origin of the MCA. Mild resistance to this advancement indicated that the intraluminal occluder had entered the anterior cerebral artery. After 60 minutes, the suture was carefully removed, until its tip was blocked by a ligature placed on common carotid artery (to allow reperfusion). The animals were placed in a cage to recover from anesthesia at room temperature and were then allowed to eat and drink ad libitum. The sham operation consisted of the same manipulation but without introduction of the monofilament.

### 2.3. Formalin Test (Pain Test)

The formalin test was carried out in a 30 × 30 × 30 cm clear plastic chamber. Behavior was rated for 1 hour from a score calculated for each minute period. 5 rats of each group were tested. Formalin (50 *μ*L) was injected subcutaneously into the plantar surface of the right hind paw while the rat was restrained manually. Behaviors were observed 3 h after the MCAO in order to be sure that animals were awaked and for 1 h. The scored behaviors were those originally described by Dubuisson and Dennis [18]: 0 = normal weight bearing the injected paw; 1 = lameness during locomotion or resting the paw lightly on the floor; 2 = elevation of the injected paw so that at most the nails touch the floor; 3 = licking or biting of the injected paw. For clarity of the results, we grouped the scores every 5 minutes for 1 hour.

### 2.4. Infarct Volume Measurement

24 hours after reperfusion, rats were killed with an overdose of pentobarbital (200 mg/kg, ip). Brains were rapidly removed and placed in ice-cold isopentane solution, frozen, and coronally dissected into 50 *μ*m thick slices on a cryostat at 12 levels in 1 mm steps, according to the Paxinos and Watson stereotaxic atlas. Sections were stained with cresyl fast violet. The unstained area of the brain was defined as the infarct zone. Infarcted cortical and subcortical areas and hemispheric areas were calculated separately for each coronal slice using image analysis software (ImageJ) after digitization.

Next, infarct volumes (total, cortical, and subcortical) and hemispheric volumes (in mm^3^) were calculated by the summing the respective areas for all sections for all animals and the distance between them. Lastly, the infarct volumes were corrected for the brain edema effect using the following equation: corrected infarct volume = total infarct volume − (left hemisphere volume/right hemisphere volume) [17].

### 2.5. Statistical Analysis

All values were expressed as mean ± standard error of the mean (SEM). Continuous variables were compared with a one-way ANOVA followed by a post hoc Tukey's Multiple Comparison Test if variance analysis was significant or with a *t*-test analysis. A value of *P* < 0.05 indicated statistical significance.

## 3. Results

### 3.1. Effect on 24-Hour Survival and Ischemic Event Rates

**Figure 2:**
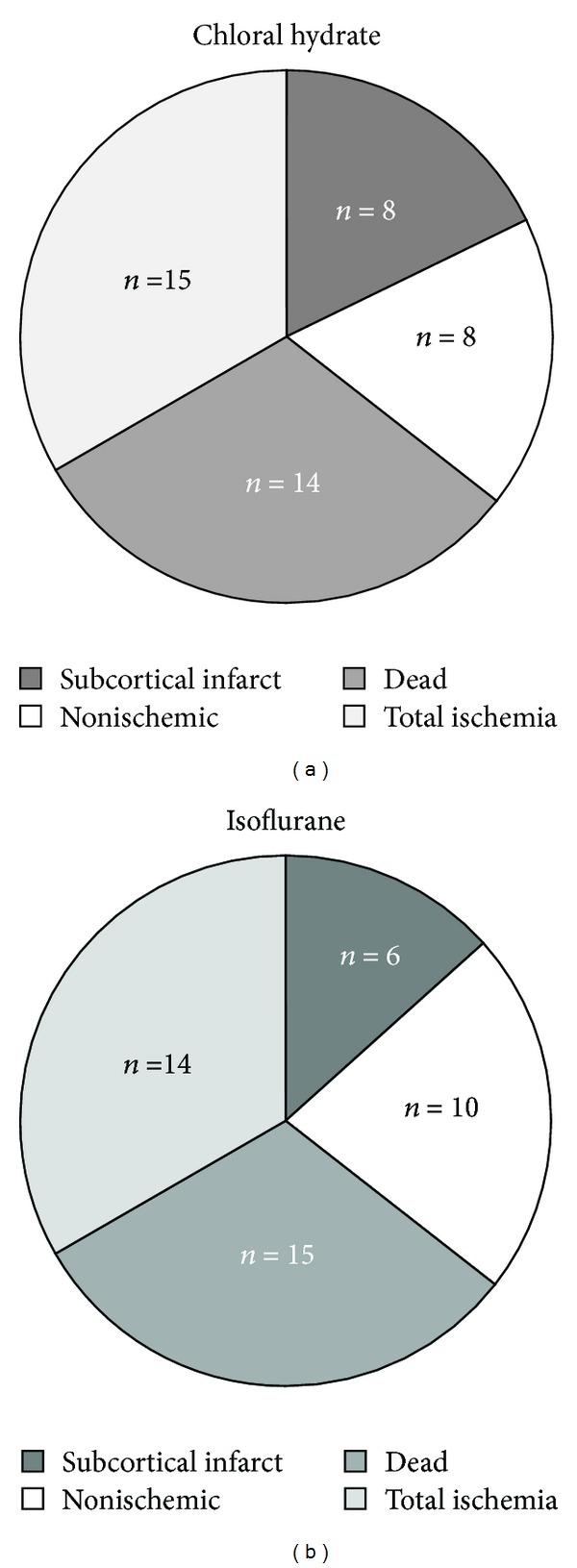
Effects of chloral hydrate and isoflurane on 24 h survival and ischemic events rates after MCAO. Frequency of infarct patterns, subcortical infarct areas and total infarct areas (ischemic), nonischemic and dead animals related to anesthesia type: (a) chloral hydrate; (b) isoflurane. *N* = 45 animals per group. Only ischemic animals were kept in the study.

Survival of ischemic animals depended on both the type of anesthesia and MK801 treatment. Thus, the death rate reached 31% in animals that were anesthetized with chloral hydrate (14/45) and 33% in anesthetized animals using isoflurane (15/45). The total ischemia rate was similar in the two groups (Figure 2).

### 3.2. Effects on Infarct Volume

**Figure 3:**
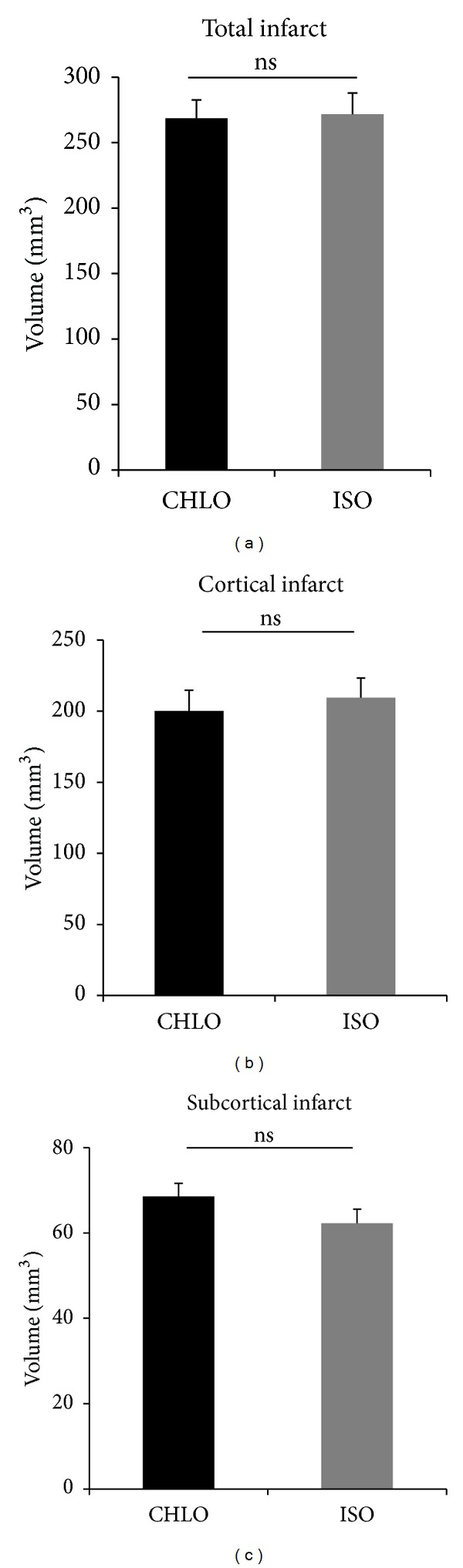
Compared effects of chloral hydrate and isoflurane on infarct volume after MCAO. Volumes are corrected from edema and expressed in mm^3^ (mean ± SEM); *n* = 8 animals per group. Differences were not significant on total infarct volume according to *t*-test analysis (*P* = 0.9862), cortical infarct (*P* = 0.7577), and subcortical infarct (*P* = 0.4079).

One-hour MCAO was followed by 23 h of reperfusion in rats according to the 2 different modes of anesthesia. The resulting infarct volumes were not different between groups, in term of total infarct volume (*P* = 0.98), cortical infarct volume (*P* = 0.75), or subcortical infarct volume (*P* = 0.41) (total: CHLO, 268.65 ± 13.93 mm^3^, ISO, 268.29 ± 14.24 mm^3^; cortical: CHLO, 200.13 ± 14.58 mm^3^, ISO, 209.51 ± 13.76 mm^3^; subcortical: CHLO, 62.27 ± 3.28 mm^3^, ISO, 68.51 ± 3.11 mm^3^) (Figure 3).

### 3.3. Compared Abilities to Modulate MK801-Induced Neuroprotection

**Figure 4:**
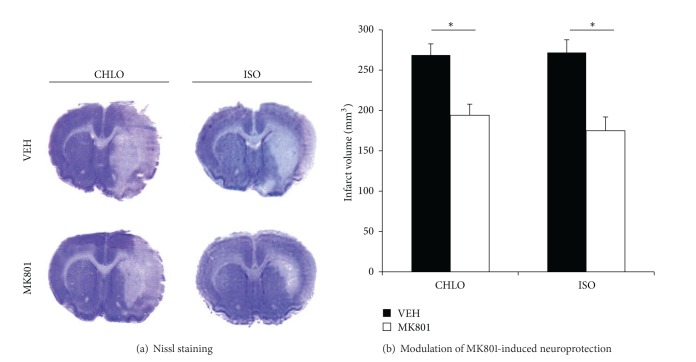
Neuroprotective effect of MK801 in rats anesthetized with chloral hydrate (CHLO) or isoflurane (ISO). (a) Representative brain slices after Nissl staining for each group treated with vehicle (VEH) or MK801 (MK801). (b) Infarct volumes (corrected from oedema) were determined after one-hour MCAO followed by 23 hours of reperfusion period. MK801 was injected three minutes before MCAO (IV, 0.5 mg/kg). Volumes are expressed in mm^3^ (mean ± SEM); *n* = 8 animals per group. Differences in infarct volume were significant according to one-way ANOVA test (*P* < 0.05).

First of all, we evaluated the neuroprotective effect of MK801 at a dose of 0.5 mg/kg IV 3 minutes before MCAO in animals anesthetized with chloral hydrate. The resulting infarct volumes obtained from animals treated with MK801 (CHLO + MK) decreased significantly in comparison with vehicle treated animals (CHLO) (CHLO: 268.65 ± 13.93 mm^3^, versus CHLO + MK: 194.10 ± 13.76 mm^3^; *P* < 0.05). Independently, MK801 induced neuroprotection in animals group anesthetized with isoflurane (ISO + MK). A significant decrease in infarct volume was found (ISO: 268.29 ± 14.24 mm^3^, versus ISO + MK: 174.98 ± 17.01 mm^3^; *P* < 0.05) (Figures 4(a) and 4(b)). To test whether isoflurane modulates MK801-induced neuroprotection, we compared neuroprotection observed in both anesthetics. No difference was observed between ISO + MK and CHLO + MK group (*P* > 0.05).

### 3.4. Compared Analgesic Abilities after MCAO

**Figure 5:**
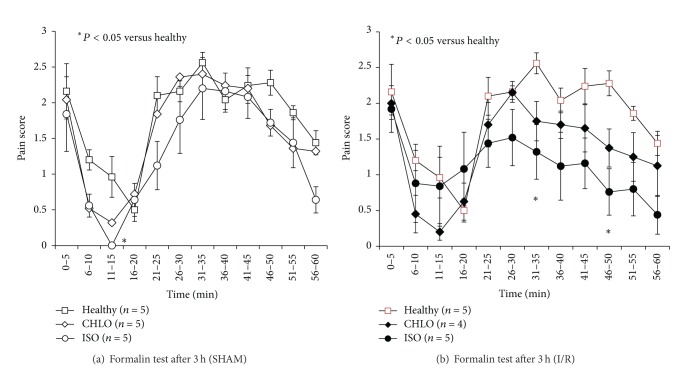
Assessment of pain after surgery including anesthesia with either chloral hydrate (CHLO) or isoflurane (ISO). Pain scores (ranging from 0 to 3) were pooled every 5 minutes. Formalin test was performed on healthy animals, sham animals, and I/R animals in each condition of anesthesia; *n* = 5 animals per group. In the SHAM group (Figure 5(a)), a significant difference was observed between isoflurane treated and healthy animals at 15 min of the formalin injection according to one-way ANOVA test (*P* = 0.02). At the other time points, no significant difference was observed between the different groups according to one-way ANOVA test. In the group I/R (Figure 5(b)), a significant difference was observed between isoflurane group and healthy animals at 35 min (*P* = 0.03) and at 50 min (*P* = 0.005) after formalin injection according to one-way ANOVA test.

To assess the analgesic effect of both anesthetic agents, we used the formalin test on animals and scored their reaction every minute for 1 hour and pooled the scores every 5 minutes. Healthy animals (HEALTHY) underwent no anesthesia and no surgery and were considered as controls for the experiment. This test was carried out 3 h after induction of anesthesia, to allow for time for all the animals to wake up and to be operational for testing. As a first step, we studied the pain felt after formalin injection for 1 hour in sham animals anesthetized with either chloral hydrate or isoflurane (Figure 5(a)). A significant difference was observed between isoflurane group and healthy animals at 15 min after formalin injection (*P* = 0.02). No significant difference was observed between isoflurane and chloral groups. In a second step, we performed the formalin test on ischemic animals (I/R) anesthetized either with chloral hydrate or isoflurane. In the same way, this test was realized 3 h after induction of anesthesia. A significant difference was observed at 35 min (*P* = 0.03) and at 50 min (*P* = 0.005) after formalin injection between isoflurane group and healthy animals (Figure 5(b)). However, no significant difference was observed between isoflurane and chloral groups.

## 4. Discussion

In this study, we wanted to study the effect of different anesthetic agents on the extent of ischemic injury. There are different classes of anesthetics used in animal testing. Among them, we can find not only injectable anesthetics, such as barbiturates, cholinergic or morphine mimetics, but also volatile anesthetics, such as nitric oxide or halogenated gas. In the literature, pentobarbital is a widely used barbiturate, but its use is delicate because it may result in a deep sleep and respiratory distress [19, 20]. The mixture ketamine-xylazine is also commonly used in MCAO studies, but it is often reported to have an inconsistent anesthetic effect, with an extended and fluctuating induction time [21]. We therefore performed MCAO in animals asleep with either chloral hydrate or isoflurane. No significant difference was observed in terms of infarct volume and mortality in different groups. In addition, we wanted to investigate the modulation of the neuroprotection induced by MK801, an antagonist of NMDA receptor, extensively studied in stroke animal models [14–16]. Whatever the anesthetic agent used here was, the infarct volume was reduced when animals were treated with MK801. As isoflurane exerts neuroprotection through additional targets other than NMDA receptor, including two-pore-domain potassium channels, and NO-mediated cerebral artery vasodilation, we expected a potentiation of neuroprotection when coadministered with MK801 [22–27]. The surprising absence of synergistic neuroprotection in the isoflurane treated animals, compared to chloral hydrate treated animals, suggests that our single dose of isoflurane can be used without interfering with early mechanisms of stroke pathophysiology such as excitotoxicity. This hypothesis is supported by the work of Sarraf-Yazdi et al. [28], who observed the same results despite the use of lower doses of isoflurane and a higher dose of MK801 on the same MCAO model in rats. This interesting property appears with much shorter anesthesia duration, and the gas nature of isoflurane enables much easier monitoring of the anesthetic process compared to injections [29].

The second part of our study aimed at evaluating the pain felt by the animal, since it is considered as an ethical problem in experimental research [30]. The formalin test is a model of somatic pain which can be achieved in animals [31, 32]. In each experiment, we obtain a biphasic response: at the early stage (possibly related to noninflammatory processes) and the late stage (surely inflammatory) [33]. This dual response occurs through the activation of different mechanisms of pain [31], the early phase one involving the stimulation of nociceptors [18] and the late phase one resulting from peripheral inflammatory processes mediated by histamine, serotonin, or prostaglandins [34]. Besides, changes in information processing take place at the spinal level during the late phase, after the afferent stimulation previously elicited in the early phase. In 1993, Field and colleagues described chloral hydrate ability to promote a sufficient anesthesia and analgesia depth for surgical procedures [35], whereas the analgesic effect of isoflurane was poorly investigated. Here, the variability found from one animal to another in the pain felt after ischemic stroke onset, appearing less intense in ischemic than in healthy animals, suggests an impact of stroke-induced damage on brain cortical and subcortical structures involved in pain feeling. Brain infarcts may modify the perception of pain since ischemic animals were less algesic than healthy animals. Although the formalin test was carried out in the nonhemiplegic limb, some adaptive or countervailing mechanisms may modify pain signal, as put forward by the *Canadian Council on Animal Care* [36]. However, the differences between both anesthetics used in this pain study were of low magnitude. Isoflurane seemed to have a slight analgesic effect, especially at the end of the first pain phase for both sham-operated and ischemic animal groups and also at the end of the second phase in the ischemic animal groups. In the literature, isoflurane affects synaptic transmission of pain and may contribute to analgesia, up to a loss of consciousness by diminishing the strength of arousing stimuli. This effect might be involved in pain prevention during anesthesia [37]. The pain felt by sham-operated animals was thus investigated in order to check whether isoflurane could keep its analgesic effect in control condition. Pain intensity was found similar in sham-operated animals to that felt by healthy animals, except for 15 minutes after the beginning of the test, where animals under isoflurane were less sensitive than the ones of the other groups. As this time point corresponds to the very end of the first phase (noninflammatory phase) and thus to the beginning of the next (inflammatory) phase, our results suggest that isoflurane may either stimulate nociceptors or act on the peripheral inflammatory processes, as reported by Liu and colleagues at the spinal level [38].

To conclude, isoflurane seems to be a good candidate for chloral hydrate replacement and use in the rat MCAO model, with additional advantages: (i) it is easy to administer and to titrate, (ii) it has a rapid onset and recovery, (iii) it produces adequate and reproducible anesthesia depth, and (iv) it causes minimal cardiac depression. Furthermore, this anesthetic agent displays a slight analgesic effect, fulfilling an important ethical criterion for animal experimentation as referred to in the guidelines of ethics committees.

## Figures and Tables

**Table 1:** Characteristics of anesthesia using chloral hydrate or isoflurane.

Agent	Administration route	Dose	Anesthesia duration	Mechanism of action
Chloral hydrate (CHLO)	Intraperitoneal	300 mg/kg	90 min	GABA A stimulation
Isoflurane (ISO)	Gas mask	Induction: 4% Maintenance: 2% Output: 2 L/min	20 min	Two-pore-domain potassium channels activation
